# Potential of apoptotic pathway-targeted cancer therapeutic research: Where do we stand?

**DOI:** 10.1038/cddis.2015.275

**Published:** 2016-01-14

**Authors:** S Baig, I Seevasant, J Mohamad, A Mukheem, H Z Huri, T Kamarul

**Affiliations:** 1 Department of Orthopaedic Surgery, Tissue Engineering Group, Faculty of Medicine, University of Malaya, Kuala Lumpur 50603, Malaysia; 2 Institute of Biological Sciences, Faculty of Science, University of Malaysia, Kuala Lumpur 50603, Malaysia; 3 Clinical Investigation Centre, University of Malaya Medical Centre, Kuala Lumpur 50603, Malaysia; 4 Department of Pharmacy, Faculty of Medicine, University of Malaya, Kuala Lumpur 50603, Malaysia

## Abstract

Underneath the intricacy of every cancer lies mysterious events that impel the tumour cell and its posterity into abnormal growth and tissue invasion. Oncogenic mutations disturb the regulatory circuits responsible for the governance of versatile cellular functions, permitting tumour cells to endure deregulated proliferation, resist to proapoptotic insults, invade and erode normal tissues and above all escape apoptosis. This disruption of apoptosis has been highly implicated in various malignancies and has been exploited as an anticancer strategy. Owing to the fact that apoptosis causes minimal inflammation and damage to the tissue, apoptotic cell death-based therapy has been the centre of attraction for the development of anticancer drugs. Increased understanding of the molecular pathways underlying apoptosis has enabled scientists to establish unique approaches targeting apoptosis pathways in cancer therapeutics. In this review, we reconnoitre the two major pathways (intrinsic and extrinsic) targeted cancer therapeutics, steering toward chief modulators of these pathways, such as B-cell lymphoma 2 protein family members (pro- and antiapoptotic), inhibitor of apoptosis proteins, and the foremost thespian of extrinsic pathway regulator, tumour necrosis factor-related apoptosis-inducing agent. Together, we also will have a look from clinical perspective to address the agents (drugs) and therapeutic strategies adopted to target these specific proteins/pathways that have entered clinical trials.

## Facts

- Hastened findings in the field of cell death and cancer have enabled us to understand the intricate molecular machinery inside of a cell, governing complex processes like cell death, and allowed us to translate those findings into promising clinical benefits
- Apoptosis or known as programmed cell death is a physiological process that is responsible for eliminating unwanted, damaged, mutated and/or aged cells that might pose robust threat to the living body if not removed. Deregulation of this pathway (excessive or recessive) is central to various diseases, cancer being one of them
- Apoptosis is triggered as a result of various DNA-damaging agents such as ultraviolet radiations and chemotherapeutic agents. In response to such stresses, a cell can activate the DNA repair systems for the damage to be repaired; however, if the damage is irreparable it will, or continue to, survive with the oncogenic mutations resulting in aberrant functions leading to various diseases like cancer.

## Open Questions

- In response to various cellular stresses, a mammalian cell is programmed to react in a number of ways. Does the nature or extent of the stress determines the type of reaction for the cell to be executed? For instance, DNA damage instigated in a cell can leave it with two choices, either to repair that damage or to instruct it to commit suicide through apoptotic pathways because the damage is irreparable. The question is who decides cell's fate or who is the decision maker? Many therapeutic agents have been proposed with robust anticancer activity capable of inducing apoptosis in cancer cells, but the mechanisms defining their mode of action remain a mystery. Further insights into that can take cancer therapeutic research in new directions
- Despite all the efforts that have been made to combat cancer we still cannot claim victory over cancer. And this really questions our research directions that have been undertaken. Are we really heading in right direction to win this battle against cancer?

Cell death is essential for life.^1^ Cell death plays critical roles in regulating embryonic development, maintaining tissue homoeostasis, controlling immune function, tumour suppression and infection resistance.^2, 3, 4^ Cell death eliminates unfit cells from the body.^5^ Throughout life, cell death must balance cell proliferation.^3^ Cell death is responsible for an array of dispensable physiological processes, including removal of useless cells from the immune system,^6^ duct formation in mammary glands, thymus degeneration with ageing and finally elimination of infected cells to limit pathogen spread.^7^ It also aids in shaping immune repertoire and refines immune responses.^4, 8^ The process responsible for execution of all of the above-mentioned events is called apoptosis. Apoptosis is perhaps the best-studied form of programmed cell death that forces the demise of useless or worn out cells. The goodness of apoptosis lies in the fact that they principally engage in dismissal of damaged or stressed cells in a fashion that is expected to cause the slightest damage and inflammation.^1, 9, 10^

Apoptotic cell death is generally characterized by a morphologically homogeneous entity.^11^ The chief morphological feature of apoptosis is shrinkage of nuclei, nuclear chromatin condensation, cytoplasmic shrinkage, dilated endoplasmic reticulum and membrane blebbing.^12^ The contents of the cell become swathed in 'apoptotic bodies' which are then recognized and engulfed by nearby phagocytic cells and digested in lysosomes.^13^ Other forms of cell death, such as necrosis and necroptosis, have been recognized and studied;^12, 14^ however, they are not addressed in this review. The fact that cell death is a genetically controlled process has allowed developments in exploring the machineries of many different diseases and has facilitated the development of pharmacological agents that initiate this cell death.

Most of what we know about apoptosis has been developed in the past few decades. These studies have unrevealed multifaceted apoptotic mechanisms that are interleaved with other significant pathways, such as cell cycle, cellular metabolic and receptor transduction pathways.^1^ In this review, we will highlight the apoptotic pathways that have been targeted by researchers for potential anticancer drug development and will discuss the current status of cell death-targeted cancer therapeutics.

## Major Signalling Pathways that Mediate Apoptosis: Distinct but Congregating

**Figure 1:**
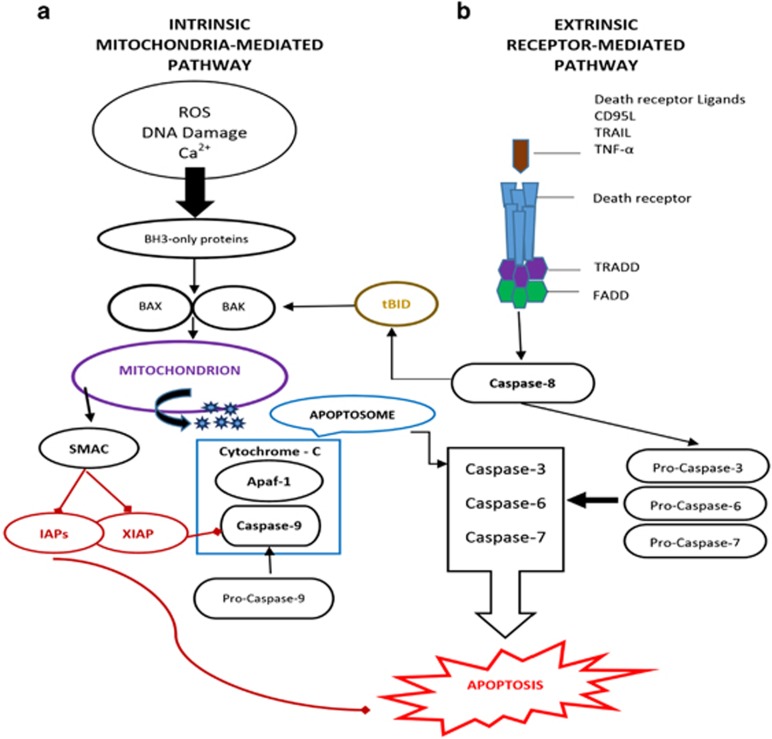
The mitochondria-mediated intrinsic (**a**) and death receptor-mediated extrinsic (**b**) pathway. Apaf-1, apoptotic protease activating factor 1; FADD, Fas-associated death domain; TRADD, TNFR-associated death domain protein

Apoptosis is triggered by chronological activation of caspase family via two distinct but congregating pathways known as intrinsic and extrinsic pathways.^15, 16^ The intrinsic pathway (commonly known as 'stress' or 'mitochondrial' pathway) is dominantly controlled by Bcl-2 protein family. This mitochondria-controlled cell death is a two-step process. At first, numerous stimuli trigger an increase in mitochondrial permeability, which results in release of apoptogenic factors through the outer membrane and disturbs the electrochemical gradient of inner membrane. This entire havoc is sensed by a multiprotein complex called mitochondrial permeability transition that resides at the junction of inner and outer mitochondrial membranes.^17^ Secondly, this mitochondrial dysfunction concludes in the disturbance of plasma membrane integrity (necrosis) and/or the activation of specific apoptogenic proteases (caspases) by mitochondrial proteins leak into the cytosol (cytochrome *c*–apoptosis-inducing factor) with activation of apoptosis.^18^ To execute apoptosis, this released cytochrome *c* requires assembling a complex called 'apoptosome' (a multiprotein caspase activating complex). Upon formation of the apoptosome complex, the key constituent of apoptosome called apoptotic protease activating factor 1 binds procaspase 9 via interface with its caspase recruitment domain^19^ and executes apoptosis. This intrinsic pathway could be initiated in response to various stresses, including DNA-damaging agents, activation of oncogenes, overload of Ca^2+^, deprivation of growth factors, oxidants and microtubule-targeted drugs (please refer to Figure 1).^20^ The mitochondrial dysfunctional consequences, such as loss of inner mitochondrial membrane potential, hyper production of superoxide ions, disturbance in mitochondrial biogenesis, outflow of matrix calcium glutathione and release of membrane proteins,^21, 22^ hold promising potential for cancer therapeutic strategies via induction of apoptosis in cancer cells which is discussed later in this review.

This mitochondrial pathway is highly controlled by BCL-2 family members that act by stimulating BH3-only family proteins which activates proapoptotic effectors BAX and BAK. These proapoptotic effectors disrupt the mitochondrial membrane that ensues in the release of cytochrome *c* that forms a complex 'apoptosome'. This complex consists of caspase-9, Apaf-1 and cytochrome *c* which activates effector caspases and executes apoptosis. The released protein second mitochondria-derived activator of caspases (SMAC) blocks the caspase inhibitor called X-linked inhibitor of apoptosis protein (XIAP). On the other hand, extrinsic/death receptor-mediated apoptosis is engaged when certain death receptor ligands, such as FAS ligand and TNF, tie up their death receptors with the plasma membrane, thereby activating caspases-8 via FADD and TRADD. These two pathways congregate at the effector caspases (caspase-3, -6 and -7). Generation of tBID by caspase-8 in death mediated pathway could engage intrinsic pathway and magnify the apoptotic response.

Alternatively, activation of these caspases is also brought about by the formation of death receptor (DR) signalling, initiated by DRs at the cellular surface.^23, 24, 25^ Initiation and execution of apoptosis via this pathway is referred to as 'extrinsic' or 'death receptor' pathway. All the members of DRs are expressed on the cell membrane and are characterized by the presence of a death domain (DD) that plays a crucial role in apoptotic signal transduction.^26^ So far, six members of DR family have been recognized: TNF-R1, CD95 (APO1/FAS), DR3, TRAIL-R1, TRAIL-R2 and DR6,^25,26^ TRAIL receptors (TRAIL-R1 and TRAIL-R2) are promising targets for cancer therapy.^27, 28, 29, 30^ This extrinsic apoptotic cell is introduced by signals originating from these cell-surface DRs activated by death ligands.^23, 31^ This triggering of DRs by death ligands ensues in the formation of a death-inducing signalling complex (DISC).^23, 32^ This DISC consists of oligomerized receptors: the DD containing adaptor molecule called Fas-associated death domain, procaspase-8 (FLICE), procaspase-10 and the cellular FLICE inhibitory proteins (c-FLIP)^33^ (Figure 1). Formation of DISC activates procaspase-8/10 and subsequently initiates proapoptotic cascade of caspases.^34^

## Carcinogenesis and Apoptosis – Molecular Targeted Therapies

One of the prominent hallmarks of cancer is evasion of apoptosis by cancer cells.^35^ Because inhibition of apoptosis lies at the heart of all tumour development, tumour cell death is required for the clearance of malignant cells and maintenance of definite number of healthy cells.^36^ Targeting cellular death pathways presents some potential targets for therapeutic intervention in all cancers.^37, 38^ The most obvious strategy for cancer therapy is to target the lesions that suppress cell death – specifically apoptosis in tumour cells. The proapoptotic effects inflicted by growth-deregulating mutations suggest that tumours depend upon the antiapoptotic factors to sustain growth. In this section, we will concentrate on the pro- and antiapoptotic proteins of BCL-2 protein family (BCL-2, BCL-xL, BCL-w, Mcl-1 and BH3-only proteins along with inhibitor of apoptosis proteins (IAPs) that have been targeted for therapeutic intervention.

## Intrinsic Pathway-Targeted Therapeutic Strategies BCL-2 Protein Family: An Introductory visit to the Family and their Therapeutic Targets

In pathological as well as physiological settings, transgenic and gene-targeted mice studies have established the function of various BCL-2 protein family members. Hence, it has been established that these proteins govern the survival of all the cells and increased expression of these antiapoptotic proteins is involved in development and progression of many tumours.^39^

Hitherto, there are a total of 25 known genes of BCL-2 protein family.^40^ These proteins dictate the cell in making cell survival/death decisions by governing mitochondrial outer membrane permeabilization (MOMP).^40, 41, 42, 43^ Members of this family are characterized by four conserved amphipathic *α*-helical regions designated BCL-2 homology (BH) 1–4 domains.^44^ Based on these domains, members of this family could broadly be categorized into three subgroups: (1) proapoptotic (multidomain) proteins BAX and BAK, (2) antiapoptotic (multidomain) proteins (BCL-2, BCL-xL, BCL-w, Bfl-1 and Mcl-1) and (3) proapoptotic (single domain) BH3-only proteins BID, BIM, BAD, p53 upregulated controller of apoptosis (PUMA) and NOXA (refer to Table 1). BH3-only proteins play a key role in regulating and promoting apoptosis and thus serve as an appealing target for therapeutic intervention.^44, 45, 46, 47, 48^

These prosurvival BCL-2 protein family members have been shown to render many cell types resistant to diverse apoptotic stimuli.^49, 50^ Interfaces between the members of the BCL-2 protein family via the BH3 unit play a critical role in regulating cell death and are central to apoptosis.^41^ BCL-2 over-expression has been found in various haematological and solid tumours, such as acute myeloid leukaemia (AML), chronic lymphocytic leukaemia (CLL), non-Hodgkin's lymphoma (NHL), myeloma, lung, breast, prostate, melanoma, hepatocellular carcinoma.^41^ Moreover, it is over-expressed as a result of t(14;18) chromosomal translocation in approximately 90% of follicular centre B-cell lymphomas.^3, 51^ Strategies that have been employed to overcome the cyto-protective effects of these antiapoptotic members include: (1) interference with mRNA function, (2) development of small-molecule drugs to target specific proteins, and (3) shut down of gene transcription. In addition to their direct role in preventing apoptotic cell death, they also act by blocking the proapoptotic proteins. For example, proapoptotic protein Bak is normally sequestered by Mcl-1 and Bcl-xL.^52^ Only when Bak is released from both Mcl-1 and Bcl-xL can it induce apoptosis.

## Antisense Oligonucleotides – Targeting the mRNA

Antisense oligonucleotides (ASOs) are short synthetic sequences of single-stranded DNA that can bind to target mRNA, ensuing inhibition of mRNA by RNase H (ubiquitous endonuclease). These ASOs function by enhancing sensitivity to cytotoxic drugs *in vitro* and xenograft models. Although, synthesis of these phosphodiester oligonucleotides is not complex matter,^53^ their use has been limited owing to the fact that are rapidly degraded by intracellular endonucleases and exonucleases.^53, 54, 55, 56^ Advancements in the development of ASOs now offer new generation ASOs with higher target affinity and stability that are being tested in clinical fashion.^57, 58^ Oblimersen sodium (G3139, Genasense), an 18-base antisense phosphorothioate oligonucleotide, is a potential anti-BCL-2 mRNA agent that has advanced in clinical settings.^40, 41^ It is the most promising inhibitor of antiapoptotic protein BCL-2 protein that has been studied for the treatment of lymphoma and is in clinical trials.^59^ It has also been tested in combination with other anticancer agents in various cancer types, such as multiple myeloma, small-cell lung cancer^60^ AML,^61^ melanoma and non-Hodgkin's lymphoma.^62^ Status of oblimersen in clinical settings is presented in Table 2.

BCL-xS, structurally similar to BCL-xL, is another antiapoptotic member. Strategies to sensitize tumour cells to chemotherapy *in vitro* using ASOs have been adopted to promote BCL-xS production. Improved efficacy of cisplatin has also been reported when used with antisense BCL-xL oligonucleotide^63^ in mesothelioma cells.

MCL-1 is another widely expressed member of the family in various haematological malignancies and solid tumours. A number of reports have endorsed its oncogenic nature.^64, 65^ Mcl-1 ASO down-regulation of MCL-1 has been identified as a potential target. MCL-1 ASO down-regulated MCL-1 significantly and synergized with ‘imatinib' in inhibiting growth of CML cells.^41^

Oblimersen is the first oligonucleotide to demonstrate proof of principle of an antisense effect in human tumours by the documented down-regulation of the target bcl-2 protein. Oblimersen provides biologically relevant plasma levels, down-regulates target bcl-2 protein within 3–5 days of initiating treatment and yields an acceptable safety profile.^66^

## Small-Molecule Inhibitors of BCL-2 Family: Regulation of Gene Expression and Interaction with Prosurvivals Histone Deacetylase Inhibitors: Regulators of Gene Expression

Histone deacetylases (HDACs) are critical controllers of gene expression.^67, 68^ They act by enzymatically removing the acetyl group from histones.^69^ Over-expression of HDACs has been established in various critical events of tumorigenesis, such as epigenetic repression of CDKN1A (encoding the cyclin-dependent kinase inhibitor p21) tumour suppressor gene and key genes, like breast cancer 1, early onset BRCA1 and ataxia telangiectasia and Rad 3 related (ATR).^67, 68^ Hence, HDACs are an attractive drug targets in oncology and inflammation.^70^ Genetic knock-down of HDACs has been shown to induce apoptosis and cell cycle arrest in a variety of tumour types, such as colon, lung, breast and acute promyelocytic leukaemia, highlighting HDAC activity as a key indicator of survival and tumorigenic capacity.^71^

**Figure 2:**
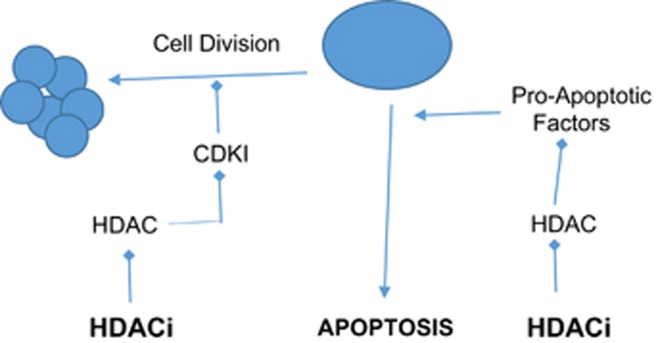
Schematic presentation of the role of HDACi

The effects of HDAC inhibitors on tumour cells include induction of tumour cell death, cell cycle arrest, senescence, differentiation, autophagy and increased tumour immunogenicity (Figure 2). The most common and the widely studied antitumor effect of HDAC inhibitors is apoptotic cell death.^72, 73^ Butyrate was the first HDAC inhibitor identified as potential anticancer therapy which was based on its ability to induce differentiation, which supports the basis that induction of differentiation of transformed cells contributes to anticancer therapies. A vast array of both natural and synthetic compounds acts as HDAC inhibitors.^71^ Two histone deacetylases inhibitors (HDACi), vorinostat and romidepsin, have been approved by FDA for the treatment against refractory cutaneous T-cell lymphoma (CTCL). Many HDACi have entered phase I to III clinical trials (refer to Table 3 for the list of currently being tested HDACi in clinical settings). More than 350 clinical trials have been completed or are underway using HDAC inhibitors, both as single agents or in combination, for the treatment of malignancies, including haematological malignancies.^123, 124^ The most successful combination therapy is the rationally designed combination of HDAC inhibition and proteasome inhibition.^125^ Two phase II clinical trials have been conducted with vorinostat and bortezomib for relapsing or refractory multiple myeloma with a response rate of 42 and 27%, respectively.^126, 127^ Another combination of vorinostat and marizomib in patients with melanoma. Despite of all the efforts that have been made for the development of HDACi, the target specificity of HDACi and the requirement for target and site selective activity to inhibit HDACs to achieve therapeutic efficacy have hindered the efforts to develop HDACi and remain a debatable issue. Another promising approach in anticancer therapy is the combination of HDAC inhibition and hormone therapy. A phase II clinical trial of vorinostat and tamoxifen was carried-out in hormone-resistant breast cancer patients with a response rate of 19%. To date, three HDAC inhibitors have been approved by the FDA for the treatments of CTCL (vorinostat (SAHA) and romidepsin (Istodax)) and peripheral T-cell lymphoma (belinostat (Beleodaq) and romidepsin),^128^ and are being further evaluated for their potential as anticancer agents – alone and in combination, in haematological malignancies and solid tumours. Table 3 represents a list of HDAC inhibitors in clinical settings.

Aberrant DNA methylation that silences the expression of tumour suppressor genes occurs recurrently in patients with AML, and an attractive target in AML is the histone methyltransferase EZH2.^74^ Trichostatin-A – an HDACi in combination with 5-AZA-CdR and DZNep – has been shown to induce a remarkable synergistic antineoplastic effect against human AML cells.^74^ Cytarabine (Ara-C) has been a major drug for AML treatment for more than three decades.^75^ Trichostatin-A – an HDACi in combination with 5-AZA-CdR and DZNep – has been shown to induce a remarkable synergistic antineoplastic effect against human AML cells.^74^ Another HDACi (valproic acid) has been shown to exhibit very promising synergism when used in combination with decitabine in AML.^76^ Vorinostat along with ACY-1215 and Belinostat are potent HDACi with a hydroxamic acid moiety targeting class I and II HDACs that has shown modest activity in multiple myelomas.^77^ Vorinostat was the first of the HDACi to be approved for clinical use in the therapy of CTCL; however, more recently, romidepsin received FDA approval for the therapy of CTCL.^78^ For the treatment of NHL, Givinostat – an orally administrated hydroxamate – is being investigated in a clinical trial.^79^ However, it has been demonstrated in a preclinical study that an intact host immune system is essential for the efficacy of HDAC inhibitors (148p) based on the evidence that immunodeficient patients would be less responsive to HDAC inhibitors (p).

## BH3 Mimetics – Misleading the Prosurvivals

There is a common agreement that BH3-only proteins are indispensable originators of apoptosis that promulgate intrinsic and extrinsic apoptotic cell death pathways. Proapoptotic members of the BCL-2 protein family (please refer to Table 1) could be further classified based on the blocks of sequence homology called BH domains. All BH3-only proteins contain only one domain in common called α-helical BH3 domain.^10^ This conserved BH3 domain has been demonstrated to play a crucial role in cancer therapy.^80, 81, 82^ This pathway is triggered by three subgroups of BCL-2 protein family: BH3 (BCL-2 homology 3)-only proteins, BCL-2, BCL-2-associated X protein (BAX) and BCL-2 antagonist/killer (BAK), which interact with each other on the mitochondrial outer membrane. BH3-only proteins serve by transmission of signals to initiate apoptosis, BH3-only proteins induced either transcriptionally or post translationally by cytotoxic stress have been shown to carry out their purpose by two mechanisms.^48, 83, 84, 85^ They act either by neutralizing antiapoptotic BCL-2 protein family^52, 86, 87^ or by directly activating BAK and BAX.^47, 88, 89, 90^ Former mode of action has been well understood both structurally and functionally and thus has been the target for drug development. They antagonize the antiapoptotic BCL-2 protein family members by binding their hydrophobic groove by the insertion of four hydrophobic residues.^91, 92, 93^ Members such as BCL-2 antagonist of cell death (BAD) and NOXA are selective in binding with its antiapoptotic siblings, whereas other BH3-only proteins, such as BIM, tBID and PUMA, neutralize all the other antiapoptotic siblings.^86, 88^

Many small-molecules BH3-mimicking agents, both natural and synthetic, have been developed^94, 95, 96, 97, 98^ (please refer to Table 4 for a list of BH3-mimicking agents). Owing to the fact that antiapoptotic BCL-2 protein family members have been successfully antagonized, the potential of these BH3 mimetic holds considerable appeal. These BH3 mimetic have been adequately demonstrated by ABT-737.^99, 100^ A list of BH3 mimetics, some of which entered clinical trials, is given in Table 4.

## Inhibitors of Apoptosis Proteins

The IAPs are the only known endogenous proteins that control the activity of both initiator and effector caspases. Controlled expression of the IAPs has been shown to influence cell death^101^ and is believed to have important consequences with respect to human cancer. Over-expression of IAPs is associated with poor prognosis and chemo-resistance in several cancers.^102, 103, 104^ IAPs are a family of proteins that serve as endogenous inhibitors of apoptosis.^105^ All these have a common domain of 70 amino-acid baculo-virus repeats (BIR) that suppress caspase function by facilitating protein–protein interactions. This allows IAPs to bind to caspases, thereby inhibiting cell death. Based on the first interrogation of the mechanism of IAPs in the laboratory of John Reeds,^106^ XIAP was found to prevent caspase-3 processing in response to caspase-8 activation, thereby inhibit the extrinsic apoptotic signalling by blocking the activity of the downstream effector caspases, as opposed to interfering directly with caspase-8 activation.^107^ In addition, cIAP1 and cIAP2 have also been demonstrated to antagonize caspase activity when co-expressed in yeast.^108^ To serve this, small-molecule inhibitors of apoptosis have been developed that act by binding to the BIR2 or BIR3 domain of XIAP, cellular inhibitor of apoptosis protein (cIAP1) and cIAP2, thereby enhancing apoptosis. The human IAP family consists of eight members: NAIP, XIAP, cIAP1, cIAP2, ILP2, survivin, livin and BRUCE.^109^

A variety of cancer cell lines and primary tumour biopsy samples show elevated IAP expression levels, including AML,^108^ renal cell carcinoma^110^ but not in non-small-cell lung carcinoma^111, 112^ or cervical carcinoma.^113^ High expression of XIAP or cIAP2 is associated with shorter overall survival, and lower complete response rates for AML.^108^ XIAP has also been identified as part of the progression signature in ovarian carcinoma^114^ and prostate cancer.^115^

IAPs have been highly exploited to be targeted in anticancer therapeutics and have been proved to be a good addition to the list of apoptosis-inducing strategies.^116^ Small-molecule inhibitors act either by targeting IAPs by mimicking SMAC (inhibitor of IAPs) or by antisense-mediated interference of XIAP mRNA and protein expression.^105^ Refer to Table 5 for the list of IAPs in clinical settings against cancer.

## Extrinsic Cell Death – the DR Pathway

Tumour necrosis factor-related apoptosis-inducing ligand (TRAIL/Apo2L) is a member of TNF family that selectively kills a diverse range of cancer cells. The members of DR family are the key players responsible for the activation of extrinsic apoptotic pathway.^117^ These members are characterized by the presence of almost 80 amino-acid long motif, termed death domain. CD95 (Fas/APO1), DR3, DR6, TNF-R1, TRAIL Receptor 1 (TRAIL-R1) and TRAIL-R2 are the major DRs. TRAIL has been shown to induce apoptosis in cancer cells via its two major cell DRs TRAIL-R1 and TRAIL-R2.^105^ Further, these have been specifically shown to be expressed at higher levels in solid tumours.^118^

Owing to its disparity toxicity for transformed as opposed to normal cells, Apo2L/TRAIL shows promise as a potential cancer therapy agent.^119^ TRAIL is probably a good candidate in cancer therapeutics.^118, 120^ Owing to the ability of TRAIL receptors of inducing cell death specifically in cancer cells, agonistic antibodies against TRAIL receptors have been developed and demonstrated to trigger apoptosis in a number of cancer cells.^121^ Despite all the success of TRAIL targeted cancer therapy, TRAIL resistance is a common impediment in TRAIL-based therapy that limits the efficacy of these drugs.^122^ Refer to Table 6 for a list of drugs targeting DRs in clinical settings.

## Conclusion

Most of what we know about apoptosis has been developed and understood only recently. In depth evaluation and assessment of this intricate machinery (cell) running inside of our bodies performing various functions has enabled us to unravel probably a bit of what is not known yet. The core idea of designing therapeutic drugs for cancer is based on the fact that worn out or damaged cells commit suicide in order for the body to continue to grow normally, maintaining a healthy number of cells, whereas this phenomenon is greatly disturbed in cancer cells. Identification of the key players involved in the execution of apoptosis and their interaction with other significant participants of apoptosis has boosted some of the significant developments made in the field of cancer therapeutics.

Apoptosis-targeted cancer therapy has been an indispensable approach in combating this deadly disease cancer; however, we are still left with huge challenges to be overcome. Development of drugs that act either by blocking the action of antiapoptotic proteins, such as IAPs, small-molecule inhibitors (antisense oligonucleotides), or by halting, hampering or interference with the transcription of RNA, such as small interfering RNA, BH3 mimetic and some HDACi, holds robust potential for use in cancer therapy; however, the hope to cure cancer is yet to be seen.

There is no qualm in the fact that our understanding of mechanistic pathways and their interaction with others has advanced considerably in last 20 years, but the fact that we have not been able to demonstrate our win in the battle against cancer questions our current strategies adopted to address cancer in near future. Responses to DNA damage have been shown to play crucial roles in responding against stresses that stimulate abnormal functions or cause DNA damage. Manipulation of this already existing mechanism could prove to be an interesting target in cancer therapeutics, owing to the fact that the major decisions of cell survival and death are decided by that response system.

## Figures and Tables

**Table 1:** BCL-2 protein family members and their function

**BCL-2 family member**	**BH domain**	**Proapoptotic**	**Antiapoptotic**	**Proapoptotic function type**
BCL-2	BH 1–4		√	None
BCL-xL	BH 1–4		√	None
BCL-w	BH 1–4		√	None
BFL-1	BH 1–4		√	None
MCL-1	BH 1–4		√	None
BAX	BH 1–3	√		Effector
BAK	BH 1–3	√		Effector
BIM	BH3-only	√		Activator
BID	BH3-only	√		Activator
BAD	BH3-only	√		Sensitizer
BIK	BH3-only	√		Sensitizer
NOXA	BH3-only	√		Sensitizer
PUMA	BH3-only	√		Sensitizer

**Table 2:** Published clinical data of Oblimersen (an antisense oligonucleotide)

**Regimen**	**Disease type**	**Phase**	**Reference**
Oblimersen	Advanced solid cancer and CLL	I and II	^105, 129^
Oblimersen with rhitoximub	NHL	II	^105, 130^
Oblimersen with mitoxantrone	CRPC	I	^105, 131^
Oblimersen with docetaxel	CRPC and breast cancer	II and I	^132, 133^
Oblimersen with docetaxel	NSCLC or SCLC	III	^40^
Oblimersen with docetaxel	HRPCa (EORTC)	II	^132^
Oblimersen with danorubicin and cytarabine	AML	I	^134^
Oblimersen with carboplatin and etoposide	SCLC	I and II	^60, 135^
Oblimersen with decarbazine	Melanoma	III	^62^
Oblimersen with dexamethasone	Advanced MM	III	^136^
Oblimersen with fludarabine and cyclophosphamide	CLL	III	^137^
Oblimersen with gemtuzumab and ozogamicin	AML	II	^138^

Abbreviations: CLL, chronic lymphocytic leukaemia; NHL, non-Hodgkin's lymphoma; CRPC, castration-resistant prostate cancer; SCLC, small-cell lung carcinoma; NSCLC, non-small-cell lung carcinoma; MM, multiple myeloma; AML, acute myeloid leukaemia

**Table 3:** List of HDAC inhibitors in clinical settings

**Agent**	**Cancer type**	**Trial phase**	**Reference**
CHR-3996	Refractory solid tumours	I	^164^
Belinostat (PXD101)	Relapsed or refractory	II	^165^
Practinostat (SB939)	Refractory solid tumours in paediatric patients	I	^166^
Practinostat (SB939) + Erlotinib	Advanced aero-digestive tract tumour	I	^167^
Entinostat (MS275) + 13-*cis* retinoic acid	Solid tumours	I	^168^
Chidamine (CS055/HBI-8000)	Solid tumours and lymphomas	II	^169^
Girinostat (ITF2357)	Relapsed or progressive multiple myeloma	II	^170^
Quisinostat (JNJ-26481585)	Advanced solid tumours	I	^171^
Panobinostat (LBH589)	Relapsed or refractory NHL and advanced solid tumours	I and II	^172, 173^
Panobinostat (LBH589) + melphalan	Relapsed or refractory multiple myeloma	I and II	^174^
Panobinostat (LBH589) + imatinib	Treatment-refractory metastatic gastrointestinal stromal tumours	I	^175^

Abbreviations: SCLC, small-cell lung carcinoma; FL, follicular lymphoma^176, 177, 178, 179^

**Table 6:** Clinical status of agonists targeting death receptors (extrinsic pathway)

**Agonist**	**Target**	**Phase**	**Reference**
Dulanermin	CRC and NSCLC	I and II	^153, 154^
**Monoclonal antibodies**	**DR agonists**		
Mapatumumab	Advanced solid tumours, and NSCLC	I and II	^155, 156, 157^
CS-1008	Advanced solid tumours	I	^158^
PR095780	Advanced solid tumours, NHL	I and II	^159^
Lexatumumab (HGS-ETR2)	Advanced solid tumours	I	^160, 161^
Conatumumab (AMG-655)	Advanced solid tumours	I	^162, 163^

Abbreviations: CRC, colorectal cancer; NSCLC, non-small-cell lung carcinoma; NHL, non-Hodgkin's lymphoma

**Table 4:** Clinical studies of BH3 mimetic as anticancer drugs

**Agents**	**Nature of the agent**	**Disease type**	**Phase**	**Reference**
Obatoclax	BH3 mimetic	SCLC and myelofibrosis	I	^139, 140^
Gossypol	BH3 mimetic	Metastic breast cancer and CRPC	I and II	^141, 142, 143^
ABT-263	BH3 mimetic	Advanced haematological cancers	I	^144^
ABT-199	BH3 mimetic	CLL	I	^145^

Abbreviations: SCLC, small-cell lung cancer; CRPC, castration-resistant prostate cancer; CLL, chronic lymphocytic leukaemia

**Table 5:** List of IAPs for the treatment of various malignancies

**Agents**	**Target disease**	**Target**	**Phase**	**Reference**
SH-130	Prostate cancer cell line	IAPs	−	^146^
JP-1201	Pancreatic cancer cell line	IAPs	−	^147^
SH-122	Prostate cancer cell line	IAPs	−	^148^
AEG35156	Advanced solid cancers	XIAP	II	^149, 150^
YM155	Advanced solid cancers	Survivin	I and II	^151^
LY2181308	CRC cell lines	Survivin	Preclinical	^152^

Abbreviations: CRC, colorectal cancer cell line; IAPs, inhibitor of apoptosis proteins

## Abbreviations

BCL-2: B-cell lymphoma 2
IAPs: inhibitor of apoptosis proteins
TRAIL: tumour necrosis factor-related apoptosis-inducing ligand
DRs: death receptors
DD: death domain
DISC: death-inducing signalling complex
c-FLIP: cellular FLICE inhibiting proteins
BID: *BH3 interacting-domain* death agonist protein
BAD: Bcl-2-associated death promoter protein
BAX: BCL-2-associated X protein
BIK: BCL-2-interacting killer protein
BCL-xL: B-cell lymphoma-extra-large protein
BH3-only proteins: BCL-2 homology 3 only proteins
cIAP: cellular inhibitor of apoptosis protein
BFL-1: BLC-2-related protein A1
MCL-1: induced myeloid leukaemia cell differentiation *protein*
*PUMA*: p53 upregulated controller of apoptosis
NAIP: neural inhibitory apoptosis protein
XIAP: X-linked inhibitor of apoptosis protein
MOMP: mitochondrial outer membrane potential
AML: acute myeloid leukaemia
CLL: chronic lymphocytic lymphoma
NHL: non-Hodgkin's lymphoma
HDACs: histone deacetylases
HDACi: histone deacetylases inhibitors
CTCL: cutaneous T-cell lymphoma
